# Parvovirus b19 infection in children with sickle cell disease, watch out for splenomegaly! A case report

**DOI:** 10.4314/ahs.v22i1.69

**Published:** 2022-03

**Authors:** Julia Alonso de la Hoz, Lucía Llorente Otones, Marta Herreros Sáenz, María José Rivero Martín

**Affiliations:** 1 Pediatrician. Pediatrics Department, University Hospital of Fuenlabrada. Madrid. Spain; 2 Pediatrics Resident. Pediatrics Department, University Hospital of Fuenlabrada. Madrid. Spain; 3 Department Head and Pediatrician. Pediatrics Department, University Hospital of Fuenlabrada. Madrid. Spain

**Keywords:** Sickle cell disease, child, human parvovirus b19, splenomegaly, aplastic anemia, splenic sequestration

## Abstract

**Background:**

Sickle cell disease (SCD) is an inherited hemoglobinopathy characterized by the presence of hemoglobin S in red blood cells. This polymerizes, distorting the red blood cells, which occlude the microcirculation and have a shorter halflife, giving rise to a chronic hemolytic anemia. This anemia is worsened by parvovirus B19, as it compromises the erythroid precursor, causing a decrease in erythrocyte production. These patients sometimes present with splenic sequestration, characterized by acute blood entrapment in the spleen, with clinical signs of hypovolemic shock. The simultaneous appearance of both leads to an extremely severe situation that requires urgent action.

**Objective:**

To describe the case of a patient with SCD and splenic sequestration, in which the suspicion of concomitant aplastic crisis affected her prognosis.

**Clinical case:**

3-year-old girl with homozygous SCD, presenting with fever, cough, vomiting and pain in the lower limbs. Upon arrival, hemodynamic instability, mucocutaneous pallor, and splenomegaly were observed. Hemogram on admission showed an acute drop in haemoglobin level with reticulocytopenia. Splenic sequestration was suspected, along with aplastic crisis, so she received a blood transfusion, subsequently showing progressive improvement. Human parvovirus B19-specific IgM and IgG antibodies were detected in the serum.

**Conclusion:**

Patients with SCD and parvovirus B19 infection must be closely observed for splenomegaly since an early identification of an enlarging spleen can lead to an early diagnosis of this complication.

## Introduction

Sickle cell disease (SCD) is an autosomal recessive genetic disease identified by the presence of sickle hemoglobin (HbS) in red blood cells^1^. Heterozygous individuals or carriers of HbS have the so-called “sickle cell trait”, a generally benign and asymptomatic condition. However, homozygotes or compound heterozygotes have a symptomatic illness with 5 different possible phenotypes^1^.

One of the highest-mortality complications is splenic sequestration^2^–^5^, defined according to the latest Spanish Society of Pediatric Hematology and Oncology (SEHOP) guide as an episode of acute splenomegaly that occurs alongside a drop in baseline hemoglobin of at least 2g/dL1. Reticulocyte counts are generally high, in response to the chronic anemization suffered by these patients.

Some viral infections can compromise erythroid precursors, causing a temporary drop in hemoglobin level that is generally mild in healthy patients^6^. However, in patients with chronic hemolytic anemia such as those with SCD, the failure of the bone marrow to respond, coupled with the short half-life of the red blood cells, causes a typically symptomatic anemia that requires a blood transfusion^6^. The most frequent cause of aplastic crises in patients with SCD is parvovirus B19 infection^2^,^6^–^9^, first described in 1981^10^. SEHOP defines an aplastic crisis as a baseline hemoglobin level that has decreased by at least 2 g/dL that occurs together with reticulocytopenia^1^.

Although it is less frequent, Parvovirus B19 can cause both complications^2^–^4^,^8^,^11^. This constitutes an extremely serious situation^3^,^4^,^8^,^12^, since it triggers severe anemia as the red blood cells get trapped into the spleen and the bone marrow can't resolve it due to the erythroid precursors are compromised.

The prognosis for these patients improves dramatically when the diagnosis is made early^3^,^4^,^8^,^12^, so it is therefore essential for physicians to be able to recognize it.

## Clinical case

3-year-old girl with homozygotic SCD (Hb SS), diagnosed with newborn screening. She was brought to the emergency room by her father with a fever of 39°C, coughing, vomiting, and pain in the lower limbs. 2 weeks before she had presented a self-limiting febrile episode that included the appearance of mouth ulcers and a blood analysis that was not indicative of drop in her baseline hemoglobin level or hemolysis, so she did not require hospitalization.

Her physical examination upon arrival revealed mucocutaneous paleness, tachycardia, sleepiness, and a noteworthy splenomegaly (spleen at 8 cm below rib edge).

Due to her hemodynamic instability, intravenous isotonic saline solution was administered at 10 mL/kg, a blood sample was drawn for analysis and cross-matching, and a first dose of intravenous antibiotic was administered (ceftriaxone, 100 mg/kg/day). The blood analysis revealed severe anemia with a hemoglobin level of 3.7 g/dL, reticulocytopenia (22,000 reticulocytes/mm^3^), and an elevated level of the enzyme lactate dehydrogenase (1097 U/L). The blood analysis and biochemistry were otherwise normal.

Due to the clinical suspicion of splenic sequestration and aplastic crisis, a red blood cell concentrate transfusion was performed, with the target hemoglobin calculated at 8 g/dL. This was completed without incident, and the normalization of the hemoglobin level was checked by a blood analysis carried out 90 minutes later, which revealed a hemoglobin count of 8.3 g/dL.

In addition, to complete the etiological study, a chest X-ray was ordered (Figure 1), which was normal, along with a serological test for the Epstein-Barr virus, cytomegalovirus, leishmaniasis, and parvovirus B19.

**Figure:**
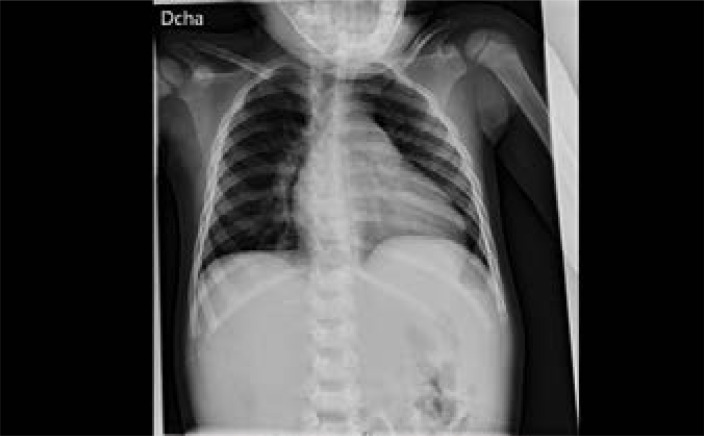


After the blood transfusion, she remained clinically and hemodynamically stable. 48 hours later, the serology results were received: the IgM and IgG were positive for parvovirus B19. Having remained afebrile, with a negative blood culture at 48 hours, a blood analysis that was not indicative of bacterial infection, and the confirmation of a recent parvovirus B19 infection, the intravenous antibiotic was discontinued and she was discharged and scheduled for a clinical follow-up 48 hours later.

On the day of her discharge, the blood analysis was repeated, which showed: stable hemoglobin (8.8 g/dL), clearly increasing reticulocytes (615,000 reticulocytes/mm^3^), and a decreasing lactate dehydrogenase level (898 U/L). The physical examination revealed a reduced spleen size (6 cm below rib edge).

3 months later, she had another episode of splenic sequestration secondary to an upper respiratory tract infection. She was thus sent to a referral hospital for splenectomy.

## Discussion

Hemoglobinopathies are detected through the newborn screenings introduced in Spain in 2004. These were first implemented in Madrid and then the practice spread to other cities in our country. Thanks to this screening, SCD is the disease that is most commonly detected at birth in several countries^1^, being an important milestone as it allows close follow-up of these patients from birth. In Spain, in 2017, there were more than 1000 people diagnosed, according to data from the national registry of hemoglobinopathies in Spain (REHem)^1^.

One of the objectives sought when working with these patients, from a very early age, is actively protecting them against complications. That´s the reason why we have to explain parents they have to go to a Pediatrician soon when children have fever, as they have more risk of developing sepsis and the reason why we prescribe broad-spectrum antibiotics when an infection is suspected.

Another important preventative measure is palpate the spleen, specially in the case of intercurrent processes, to recognize a splenic sequestration as soon as possible, due to it can cause hypovolemic shock with multi-organ failure4 and even death, as is reported by Wethers et al^3^ or Saad et al^8^. This complication is more frequent in small children^4^, like our patient, in patients treated with hidroxyurea^5^ and in those with less severe phenotypes (SC, Sβ+)^13^, since the spleen is preserved for longer.

As Brousse et al reported^5^, increased inflammation during a febrile condition may promote the trapping of blood within the spleen, causing a massive entrapment of red blood cells, resulting in a splenic sequestration. Parvovirus B19 is a well-known cause of fever, being isolated in some children with SCD and splenic sequestration^2^,^4^,^8^,^11^. Furthermore, this virus characteristically produces aplastic crisis in these patients^6^,^7^,^9^–^11^, so they are more likely to suffer both complications simultaneously, developing severe anemia with poor prognosis if red blood cell transfusion is not performed^2^,^4^,^8^.

In other hand, we usually find reticulocytopenia in these children, as in our patient, when a high reticulocyte count is expected due to splenic sequestration.

Splenectomy may be considered when patients have suffered 2 or more splenic sequestration^1^,^5^, as in our case.

## Conclusion

Parvovirus B19 can trigger more than one complication at a time, increasing its morbidity and mortality. One of the most serious situation is the appearance of an aplastic crisis along with splenic sequestration, so we should bear this complication in mind. Moreover, it´s essential to look for an enlarging spleen in SCD patients as its early detection substantially improves the prognosis.

## Figures and Tables

**Table 1:** Clinical and laboratory parameters

	At admission	90 minutes after red blood cell transfusion	First day after admission	On discharge	48 hours after discharge
Spleen size (cm below rib edge)	8	---	6,5	6	1–2
Hemoglobin value (g/dl)	3,7	8,3	---	8,8	10,2
Reticulocyte count (reticulocytes/mm^3^)	22.000	73.000	---	615.000	158.000
Enzyme lactate dehydrogenase level (U/L)	1097	---	---	898	511
